# Optimization of Bicomponent Electrospun Fibers for Therapeutic Use: Post-Treatments to Improve Chemical and Biological Stability

**DOI:** 10.3390/jfb8040047

**Published:** 2017-10-16

**Authors:** Antonio Papa, Vincenzo Guarino, Valentina Cirillo, Olimpia Oliviero, Luigi Ambrosio

**Affiliations:** 1 Institute for Polymers, Composites and Biomaterials, National Research Council of Italy, Mostra d’Oltremare, Pad. 20, V. le Kennedy 54, 80125 Naples, Italy; antonio.papa1984@yahoo.it (A.P.); valentina.cirillo@unina.it (V.C.); olivier@unina.it (O.O.); ambrosio@unina.it (L.A.); 2 IMAST Scarl, P.za Bovio 22, 80133 Naples, Italy

**Keywords:** electrospinning, gelatin, crosslinking, hMSC, drug release

## Abstract

Bicomponent electrospun nanofibers based on the combination of synthetic (i.e., aliphatic polyesters such as polycaprolactone (PCL)) and natural proteins (i.e., gelatin) have been extensively investigated as temporary platforms to instruct cells by the release of molecular/pharmaceutical signals for the regeneration of several tissues. Here, water soluble proteins (i.e., gelatin), strictly embedded to PCL, act as carriers of bioactive molecules, thus improving bioavailability and supporting cell activities during in vitro regeneration. However, these proteins are rapidly digested by enzymes, locally produced by many different cell types, both in vitro and in vivo, with significant drawbacks in the control of molecular release. Hence, we have investigated three post-processing strategies based on the use of different crosslinking agents—(1-ethyl-3-(3-dimethylaminopropyl)carbodiimide hydrochloride) (EDC), glyceraldehyde (GC), and 1,4-butanediol diglycidyl ether (BDDGE)—to delay the dissolution time of gelatin macromolecules from bicomponent fibers. All of the qualitative (i.e., SEM, TGA) and quantitative (i.e., Trinitrobenzene sulfonate (TNBS) and bicinchoninic acid (BCA) assays) morphological/chemical analyses as well as biocompatibility assays indicate that EDC crosslinking improves the chemical stability of bicomponent fibers at 37 °C and provides a more efficient encapsulation and controlled sustained release of drug, thus resulting in the best post-treatment to design bio-inspired fibrous platforms for the extended in vitro release of drugs.

## 1. Introduction

The application of micro- and nanofibers as molecular carriers is currently gaining attention for the design of drug delivery systems (DDSs), due to several advantages including improved therapeutic index, localized delivery, and reduced toxicity of drugs [1]. Indeed, their high surface-to-volume ratio and other surface characteristics (i.e., surface roughness, porosity, etc.) may drastically influence their ability to incorporate a wide range of drugs as well as to dissolve by controlled rates, thus leading to a more efficient release mechanism [2]. Meanwhile, their interconnected porous structure, offered by the random organization of fibers in the 3D network, finely mimics the native extracellular ECM-like architecture, thus assuring a full in vitro permeability to small molecules [3].

In this context, the development of electrospinning nanotechnology offers a unique opportunity for the fabrication of fibrous carriers from a large variety of synthetic [4] or natural polymers [5], for a fast, sustained, or delayed release of different kinds of molecules (i.e., drugs, enzymes, bioactive fragments) [6,7]. Moreover, electrospinning allows incorporating drug or active compounds such as growth factors into fibers, preserving them from light-driven degradation mechanisms by the proper configuration of the process setups [8]. Besides, different DDSs may be designed as a function of the peculiar drug release profile, occurring via diffusion alone or diffusion and scaffold degradation, thus providing a one-shot, sustained, or site-specific delivery of drugs to the body in response to clinical or therapeutic demands [9,10].

Currently, one of the main challenges is to control the extended burst release of hydrophilic drugs generally loaded in monocomponent electrospun polymeric fibers. Indeed, the molecules adsorbed on the surfaces of electrospun fibers may be rapidly released in the local microenvironment, resulting in a burst release at the initial stage of drug delivery [11]. Indeed, the rapid solubility of drugs induces the body to quickly absorb and metabolize them through the processes of dissolution, thus making it difficult to achieve stable long-term release and, therefore, ideal therapeutic effects [12]. In order to improve the clinical effect of water-soluble drugs, electrospun carriers have to be properly designed by including bioactive phases in different forms (i.e., blends, nanoparticles, micelles, or liposomes) able to carry molecular species in different ways. Among them, an interesting strategy consists of the fabrication of multicomponent fibers obtained by the combination of different polymers—i.e., synthetic ones with good processability and good mechanical properties, as well as natural polymers able to increase cellular attachment and biocompatibility [13]. Recently, several studies have investigated how the use of bicomponent electrospun fibers combining biodegradable polyesters (i.e., poly-ε-caprolactone (PCL)) with naturally derived polymers (i.e., collagen, gelatin) may overcome some limitations of monocomponent fibers mainly related to rapid drug release and the low efficiency of drug loading [14,15]. Indeed, the blending of bioactive proteins into synthetic electrospun fibers reduces the gap in biodegradation and biocompatibility properties with respect to natural tissues, thus resulting in very promising instructive scaffolds for controlled release applications [16]. The high biocompatibility of these proteins has been largely studied, proving their ability to promote many integrin binding sites for cell adhesion, differentiation, and mineralization [17,18]. Moreover, chemically embedded gelatin to fibers may be suitable to design bio-recognized polymer carriers able to efficiently deliver molecular species in in vitro microenvironment. This is due to the peculiar mechanism of release, mainly driven by protein depletion mechanisms in water, able to passively deliver molecular species, previously entrapped into the fibers.

Hence, we prove that gelatin stability in vitro may be improved using crosslinking fiber treatments which allow delaying the release kinetics of drugs, extending their use for a large set of therapeutic applications. For this purpose, we have optimized different crosslinking post-treatments—based on the use of three chemical agents, (1-ethyl-3-(3-dimethylaminopropyl)carbodiimide hydrochloride) (EDC), glyceraldehyde (GC) and 1,4-butanediol diglycidyl ether (BDDGE) respectively, for the study of diclofenac loaded electrospun PCL/gelatin fibers.

## 2. Materials and Methods

**Figure 1:**
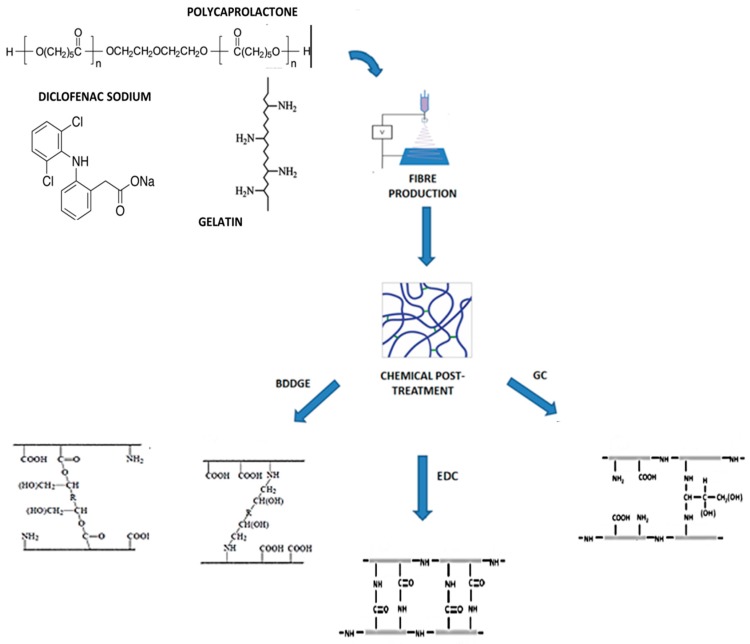
Schematic diagram of the production process: polycaprolactone/gelatin fibers were fabricated by the electrospinning technique. The diclofenac was loaded into the fibers during the electrospinning process. Successively, fibers were treated with three different crosslinking agents.

The preparation of the fibers has been efficaciously figured by a two-step process (Figure 1) involving fibers fabrication by electrospinning and chemical crosslinking of protein.

## 3. Fiber Fabrication

PCL (Mn = 45 kDa), gelatin type B (~225 Bloom), 1,1,1,3,3,3-hexafluoropropanol (HFIP), and sodium diclofenac were purchased from Sigma-Aldrich. To prepare polymer solutions suitable for electrospinning, PCL and gelatin were separately dissolved in HFIP at a polymer concentration of 0.1 g/mL. The solutions were kept under magnetic stirring at room temperature for 24 h. As the complete dissolution was reached, diclofenac—5 wt % with respect to polymers—was mixed to obtain a homogeneous solution. The PCL/gelatin 50:50 mass ratio was chosen to produce electrospun fibers [19]. Briefly, the electrospinning process was carried out by using commercial equipment (NANON 01, MECC, Fukuoka, Japan). A constant volume flow rate was maintained at 0.5 mL/h while the high voltage applied to the spinneret was 13 kV. The distance between the 18 G needle and the grounded target was 12 cm. Randomly dispersed fibers were collected on an aluminum foil for 1 h.

### 3.1. Fiber Post-Treatments

Three crosslinking methods were examined in this study:

(a) **Crosslinking with EDC**. Crosslinking was carried out in 2-(*N*-morpholino)ethanesulfonic acid (MES) buffer containing a mixture of EDC and *N*-hydroxysulfosuccinimide (N-NHS, molar ratio of EDC/NHS = 2). The amount of EDC was set in order to maintain the molar ratio of EDC to carboxylic groups of gelatin at about 2 [20]. The reaction time and temperature were set at 19 h and 4 °C. After the removal of the crosslinking solution, samples were washed three times with phosphate buffer (0.1 M, pH = 7.4) and dried on a Teflon plate.
(b) **Crosslinking with GC**. GC was dissolved in a 70% (v/v) ethanol/water solution for 1 h with a concentration of 0.5% w/w, as reported by Sisson et al. [21], and 1 mL of crosslinking solution was used on each disc. The reaction was carried out for 19 h at room temperature. At the end of the reaction, each disc was washed three times with phosphate buffer (0.1 M, pH = 7.4) and dried on a Teflon plate.
(c) **Crosslinking with BDDGE**. The crosslinking solution was prepared dissolving the BDDGE in ethanol with a concentration of 6.2% w/w. The reaction was carried out with 1 mL of crosslinking solution for each disc for seven days at 37 °C, as reported by Fiorani et al. [22]. At the end of the reaction, each disc was washed three times with phosphate buffer (0.1 M, pH = 7.4) and dried on a Teflon plate.

### 3.2. Chemical/Physical Characterization

(a) **Crosslinking degree measurements**. The degree of crosslinking was quantified using a 2,4,6-trinitrobenzene sulfonic acid (TNBS) assay. Briefly, each specimen with a circular shape—8 mm in diameter—was weighed and 1 mL of 0.5% w/v TNBS solution and 1 mL of 4% w/v sodium hydrogen carbonate (NaHCO_3_, pH 8.5) were added. The solution was heated at 40 °C for 2 h. Termination of reaction was achieved by the addition of 2 mL of 6 M hydrogen chloride (HCl) and the incubation time was continued at 60 °C for 90 min. The absorbance of the solutions was determined spectrophotometrically at 405 nm. The degree of crosslinking was calculated from the difference in the absorbance divided by the absorbance of uncrosslinked discs normalized for the weight.
(b) **Differential scanning calorimetry (DSC)**, (TA Instruments mod. TA2910, Milan, Italy). Fibers’ thermal proprieties were measured by DSC. Analyses were carried out under a nitrogen atmosphere, by heating the sample from 25 to 200 °C at 10 °C min^−1^.
(c) **Morphology**. The morphology of PCL/gelatin fibers was qualitatively estimated before and after crosslinking treatment by field emission scanning electron microscopy (FESEM, QUANTA200, FEI, Eindhoven, The Netherlands). Samples were dried in the fume hood for 24 h, mounted on metal stubs and sputter-coated with gold palladium. SEM images were taken under high vacuum conditions using the secondary electron detector (SED).

## 4. Degradation Studies and Release

In order to evaluate the efficacy of the different crosslinking methods, PCL/gelatin fibers were incubated at 37 °C in deionized water for 4, 24, 72, and 168 h. Morphological and thermogravimetric (TG) analyses were performed on the fibers to estimate the gelatin content after chemical post-treatment, at the end of the incubation period. Otherwise, supernatants were used for the bicinchoninic acid (BCA) assay. Weight loss measurements were performed under a nitrogen atmosphere from 40 to 600 °C at 10 °C min^−1^ (TA Instruments, Q500, New Castle, DE, USA). Gelatin in crosslinked membranes was detected as the weight loss ratio occurring around 300 °C. Thermo Scientific Pierce BCA Protein Assay is a colorimetric method used to quantify the total amount of protein. This assay combines the well-known reduction of Cu^2+^ and Cu^1+^ by a protein in an alkaline medium with the highly sensitive and selective colorimetric detection of the cuprous cation using a unique reagent containing BCA. The purple-colored reaction product is a water soluble complex and exhibits a strong absorbance at 562 nm that is linearly correlated with increasing the protein concentrations over a broad working range (20–2000 μg/mL). Briefly, 200 μL of working reagent was added to 50 μL of supernatant coming from the degradation tests, and incubated in a multi-well plate for 30 min at 37 °C. After cooling the plate at room temperature, the absorbance was measured at 562 nm to quantify the amount of gelatin released during the degradation treatments.

### 4.1. In Vitro Drug Release

One gram of the nanofibers of PCL/gelatin/diclofenac membranes was first soaked into 10 mL of a phosphate buffer solution PBS, and the diclofenac release studies were carried about at 37 °C and 100 rpm in a thermostatic shaking incubator. From the buffer solution, samples were taken at different times until 15 days of release for the spectroscopic analysis. The amount of released drug was determined by UV-vis spectroscopy (Pelkin Elmer, Victor X3, Milan, Italy) at 280 nm, using a calibration curve constructed from a series of DicNa solutions with standard concentrations. The experiments were performed in triplicate with freshly prepared drug-loaded fiber mats for each set.

### 4.2. In Vitro Culture

Human Mesenchymal Stem cells (hMSCs) (Clonetics, Milan, Italy) were cultured in α-Modified Eagle’s medium (α-MEM) containing 10% (v/v) FBS, 100 U mL^−1^ penicillin, and 0.1 mg mL^−1^ streptomycin, in a humidified atmosphere at 37 °C and 5% CO_2_. After post-treatment, bicomponent fibers were soaked in a solution of ethanol-PEN/STREP (70:30 v/v) as a bland sterilization strategy for cell-culture experiments. Then, 2 × 10^4^ cells suspended in 20 µL of medium were statically seeded onto the scaffold. Cell viability and proliferation were evaluated by using the Alamar Blue assay. The cell-scaffold constructs were removed from the culture plates at days 1, 7, and 14, washed with PBS, and placed into 24-well culture plates. For each construct, 2 mL of DMEM medium without Phenol Red containing 10% (v/v) Alamar Blue (AbD Serotec Ltd., Kidlintong, UK) was added, followed by incubation for 4 h at 37 °C and 5% CO_2_. The solution was subsequently removed from the wells and analyzed by a spectrophotometer at wavelengths of 570 and 600 nm.

## 5. Results and Discussion

**Figure 2:**
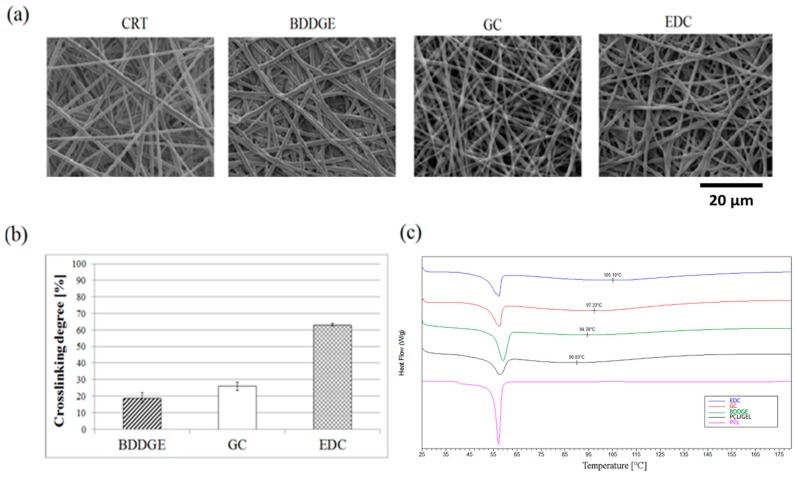
(**a**) SEM images of electrospun PCL/gelatin before the crosslinking treatments (CRT) and after the treatment with different crosslinkers; (**b**) Degree of crosslinking of electrospun PCL/gelatin fibers; (**c**) Differential Scanning Calorimetry (DSC) curves of bicomponent PCL/gelatin fibers after different post-treatments. PCL fibers were used as a negative control.

**Figure 3:**
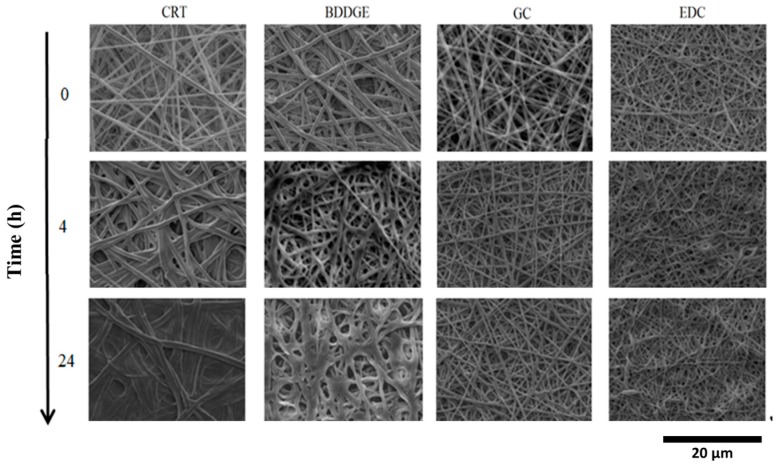
SEM microstructures of uncrosslinked (CRT) and crosslinked (BDDGE, GC, and EDC) electrospun bicomponent fibers conditioned in bi-distilled water at 37 °C for 24 h.

Herein, three different chemical post-treatments were investigated in order to improve the chemical stability of bicomponent fibers fabricated by electrospinning from a homogeneous solution of PCL and gelatin in HFIP. SEM images of bicomponent fibers after BDDGE, GC, and EDC treatment are shown in Figure 2a. After the crosslinking reaction, electrospun fibers seem to preserve their integrity and random network organization, only showing isolated beads along fibers. A slight reduction of fiber diameter may be ascribable to the effect of GC and ECD treatment (Table 1—first line). Contrariwise, EDC and GC crosslinking agents do not significantly influence fiber morphology after degradation (24 h) at 37 °C (Figure 3). Only in the case of BDDGE treatment (Figure 2a) did crosslinked fibers show a partial modification of their morphology, with a reduction of fiber diameter homogeneity, with respect to untreated fibers, used as a control (CRT). In particular, bicomponent fibers appear more flattened, probably due to the slower reaction rate of BDDGE that is able to promote a faster depletion of surface protein. Besides, this result is also affected by the used solvent—i.e., ethanol—able to promote a macroscopic swelling of gelatin phases during the treatment. Contrariwise, this effect is drastically reduced in the case of GC treatment—as a consequence of the ethanol dilution in water—and becomes completely absent in the case of EDC treatment—due to the use of MES solution, as reported in previous experimental studies [23,24]. Accordingly, TNBS assays—used to evaluate the extent of crosslinking reaction (Figure 2b)—showed the highest crosslinking degree (63 ± 0.8%) in the case of EDC treatment, down to 30 ± 2.5% and 20 ± 3% in the case of GC and BDDGE treatments, respectively. Besides, this is a consequence of the highest reactivity of EDC with respect to GC and BDDGE. Despite the crosslinking reaction being triggered by the local interaction among amino-groups, the average size of the crosslinking agents may drastically influence the reaction advancement [25]. As for the crosslinking agents used, GC macromolecules are smaller than BDDGE ones, thus allowing for a more efficient penetration into the fiber mesh. Moreover, the ability of BDDGE to simultaneously bind even two carboxylic groups may also partially inhibit the binding with amide groups, strictly requested in gelatin crosslinking, thus further reducing the crosslinking degree (Figure 2b).

These results are in agreement with data obtained by calorimetric analyses. Figure 2c shows a comparative analysis of DSC thermogram curves from bicomponent fibers after different post-treatments. In this case, uncrosslinked PCL and PCL/gelatin fibers are reported as controls. In detail, bicomponent fibers show two endothermic peaks: the first one at 58 °C is associated with the melting heat of PCL, and the less sharp one at 90 °C is ascribable to gelatin phase and related to gel transition occurring at 37 °C, as reported in literature [16]. Thermogram curves clearly indicate a shift of the gelatin peak to higher temperature values as a function of the crosslinking degree of fibers. This shift is less evident—up to 94 °C—in the case of BDDGE treatment, while it is more pronounced—up to 97 °C—in the case of GC post-treatment, and strongly marked—up to 105 °C—in the case of EDC post-treatment, according to previous trends obtained by crosslinking degree measurements (Figure 2b). It is important to underline the fact that thermal transitions are strictly affected by the presence of water molecules adsorbed in the amorphous gelatin phases along fibers. Hence, the right shift of the gelatin peak can be considered as an indicator of protein stability along fibers, being directly related to the difficulty of removing water molecules from fibers.

In order to further prove the contribution of the specific post-treatment used, gelatin degradation was qualitatively analyzed in terms of changes in fiber morphology into bi-distilled water at 37 °C at different times—4, 24, 72, and 168 h, respectively. As expected, the morphology of GC- and EDC-treated fibers did not significantly change after first 24 h (Figure 3) in contrast with the case of BDDGE-treated or untreated fibers, where a faster dissolution of gelatin occurred after just few hours under the same conditions.

**Figure 4:**
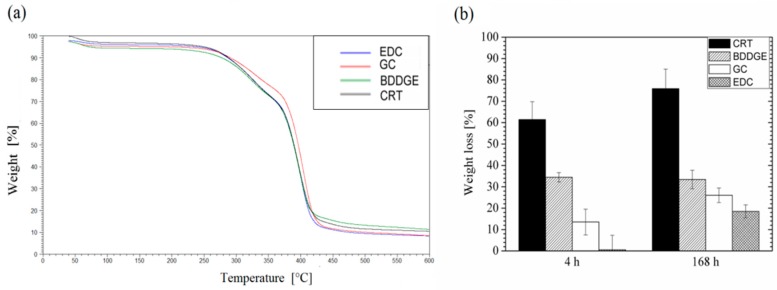
Termogravimetric analyses of bicomponent fibers after different post-treatments: (**a**) thermogram curves and (**b**) gelatin weight loss (%) from bicomponent fibers after 4 h and 168 h in bi-distilled water at 37 °C, calculated as weight loss from the thermogram curves.

The effect of chemical post-treatment on fiber morphology was firstly quantified during degradation in terms of fiber diameter variation by image analysis (see Table 1). In the case of untreated samples, an increase of fiber diameter may be recognized, due to the swelling of gelatin phases, mainly onto the fiber surface. Contrariwise, in the case of treated samples, fiber diameter is smaller at baseline with respect to the control, probably due to a partial removal of gelatin macromolecules during the crosslinking treatment. Significant variation of fiber diameter cannot be recognized among samples across time. This effect has been further investigated via TGA analysis by a quantitative measurement of gelatin weight loss (GL) from fiber mats during their incubation at 37 °C (Figure 4). In all the reported curves, percentage weight decay occurs at 320 °C, related to the thermal degradation of proteins, but relevant differences may be detected as a function of the specific post-treatment. In the case of untreated bicomponent fibers, GL is equal to 61% after 4 h, reaching 80% after 168 h. In the case of crosslinked fibers, GL is equal to 38% and 40% after 4 and 168 h; for BDDGE treatment, it is only equal to 10% and 28% for GC treatment, and equal to 2% and 20% for EDC treatment, after 4 and 168 h respectively, according to values of the crosslinking degree previously recorded in Figure 2c.

**Figure 5:**
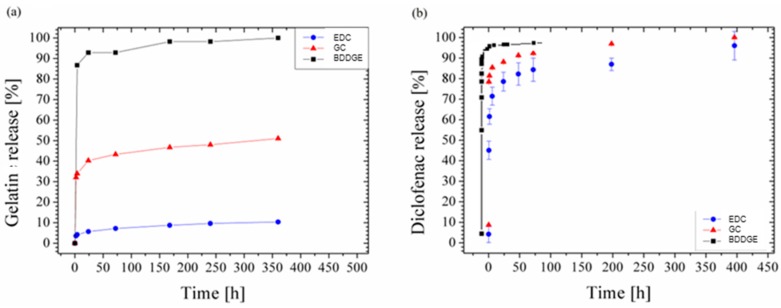
Release percentage of gelatin (**a**) and diclofenac (**b**) from electrospun bicomponent fibers treated with different crosslinkers.

All the previous data clearly show that fiber post-treatment by different chemical agents mainly influences the protein dissolution kinetic, thus suggesting the use of chemical post-treatment of bicomponent fibers to better control molecular release. In order to validate these hypotheses, electrospun bicomponent fibers were loaded with sodium diclofenac, and used as model drug in this study. The BCA test was preliminary performed to quantify the kinetic release of gelatin macromolecules (Figure 5a) for 360 h in buffered medium. According to previous data reported in Figure 3, BCA tests show a fast and complete release of gelatin macromolecules in the case of BDGGE after just few hours, while no complete dissolution of fibers—resulting in a protein release equal to 50% and 10% after 360 h—is detected in the case of fibers after GC and EDC post-treatments.

Figure 5b reports the diclofenac release from fibers in the case of bicomponent fibers after different post-treatments. Accordingly, all of the curves show an initial burst region followed by a sustained drug release. Different bursts were detected as a function of the specific post-treatment used. In particular, as the crosslinking degree increases, intermolecular forces rise up, thus resulting in a more drastic reduction of the amount of diclofenac released along the burst region. Besides, the release mechanism from similar systems may be commonly given by two subsequent events, basically driven by water diffusion through amorphous (i.e., gelatin) and crystalline (i.e., PCL) phases, both concurring with the protein depletion, but at different times. However, in this case, we consider the assumption that drug release might proceed mainly by diffusion through amorphous phases, and their dissolution may be affected only by different crosslinking degrees.

**Figure 6:**
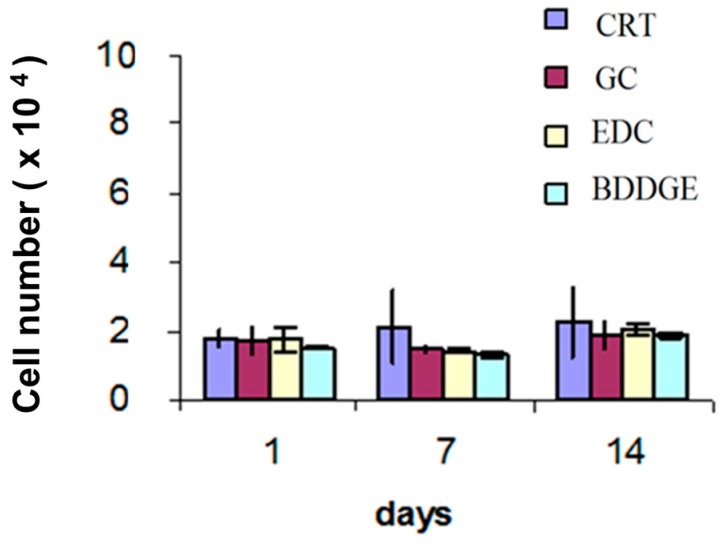
Cytotoxicity test by Alamar Blue on hMSCs culture onto diclofenac loaded PCL/gelatin fibers after different post-treatments.

In order to validate drug-loaded bicomponent electrospun fibers for biological use, we preliminary investigated the cytotoxic effect of fiber post-treatments on the in vitro response of hMSCs, potentially due to the undesired release of unreacted crosslinking agents. In this work, cytotoxicity tests were extended for 14 days, also giving information about the contribution of gelatin and drug release to in vitro hMSCs activity. Figure 6 shows the proliferation of hMSCs estimated via Alamar Blue assay, providing a quantitative evaluation of viable cells onto the scaffold. hMSCs vary from 2 × 10^4^ at day 1 to 2.4 × 10^4^ at day 14, with insignificant differences as a function of the fiber post-treatment used. Accordingly, seeding efficiency, evaluated as the difference between cells number in the seeding suspension and cells in the culture plate after seeding, is about 90%. In all cases, no statistically significant differences were recognized for different culture times among specimen populations, thus confirming that no significant toxic effect is related to the presence of crosslinking agent traces up to 14 days in culture.

## 6. Conclusions

Herein, we have validated the use of chemical post-treatments to improve the chemical, physical, and biological stability of bicomponent fibers in vitro. Three different methods have been examined to crosslink gelatin proteins into PCL fibers after the fiber fabrication via electrospinning. All methodologies based on the use of EDC, GC, and BDDGE, respectively, reached good fiber stability at 37 °C for seven days. Comparative studies have demonstrated that EDC treatment is the most efficient to slow down the depletion of gelatin macromolecules from fibers, thus extending in time the release of stable drugs such as sodium diclofenac, used in this study. Follow-up studies did not indicate significant effects of post-treatments on the in vitro response of hMSC in terms of cytotoxicity, thus confirming EDC post-treatment as an efficient method to improve the in vitro stability of drug-loaded fibers for therapeutic applications.

## Figures and Tables

**Table 1:** Variation of fiber diameter as a function of the degradation time at 37 °C.

Time (h)	CRT (µm)	BDDGE (µm)	GC (µm)	EDC (µm)
0	1.2 ± 0.3	1.2 ± 0.4	1.0 ± 0.3	0.9 ± 0.3
4	1.7 ± 0.5	1.1 ± 0.3	0.8 ± 0.2	0.9 ± 0.2
24	n.a.	n.a.	0.9 ± 0.3	0.9 ± 0.4
72	n.a.	n.a.	1.0 ± 0.5	0.7 ± 0.3
168	n.a.	n.a.	0.9 ± 0.4	0.7 ± 0.2
